# Adaptation and validation of moral distress thermometer in Chinese nurses

**DOI:** 10.1186/s12912-024-02127-0

**Published:** 2024-07-04

**Authors:** Xu Tian, Xiuni Gan, Yi Ren, Feili Li, Maria F. Jimenez Herrera, Fangrong Liu

**Affiliations:** 1 Division of Science & Technology and Foreign Affairs, Chongqing Traditional Chinese Medicine Hospital, Chongqing, 400020 China; 2 Department of Nursing, The Second Affiliated Hospital of Chongqing Medical University, Chongqing, 400010 China; 3 Department of Classic TCM, Chongqing Traditional Chinese Medicine Hospital, No.6, 7th Branch Road of Panxi Road, Jiangbei District, Chongqing, 400020 China; 4 Department of Nursing, Chongqing Traditional Chinese Medicine Hospital, Chongqing, 400020 China; 5 Nursing Department, Universitat Rovira I Virgili, Tarragona, 43002 Spain; 6 Department of Outpatient, Chongqing University Cancer Hospital, No. 181, Hanyu Road, Shapingba District, Chongqing, 400030 China

**Keywords:** Moral distress, Moral distress scale-revised, Moral distress thermometer, Registered nurses, Cultural adaptation

## Abstract

**Background:**

Moral distress seriously affects professional nurses, and a number of instruments have been developed to measure the level of moral distress. The moral distress thermometer (MDT) is one of the commonly used instruments that can rapidly measure real-time moral distress; however, it remains unclear whether it is still useful in the Chinese cultural context.

**Aim:**

This study aimed to adapt and validate the MDT among Chinese registered nurses.

**Research design:**

An online, cross-sectional, survey study of adapting and validating Chinese version of MDT.

**Participants and research context:**

A total of 182 registered nurses effectively finished this survey. The correlation between MDT score and the score of the moral distress scale-revised version (MDS-R) was used for evaluating convergent validity, and MDT scores of registered nurses who working in different departments and who made different actions to the final question of the MDS-R were compared by using one-way ANOVA to evaluate construct validity.

**Ethical considerations:**

The Ethics Committee of Chongqing Traditional Chinese Medicine Hospital approved this study.

**Results:**

The Chinese version of MDT was described as relevant to measure moral distress, with a reported item-level content validity index (I-CVI) and scale-level CVI (S-CVI) of 1. The mean MDT score and mean MDS-R score were 2.54 and 38.66, respectively, and the correlation between these two scores was significantly moderate (*r* = 0.41). Nurses working different departments reported different levels of moral distress, and those working in intensive care unit reported the highest level of moral distress than those working in other departments (*p* = 0.04). The MDT scores between nurses who presented different actions to their position were also significantly different, and those who had ever left and those who had considered leaving but did not leave reported significantly higher moral distress.

**Conclusion:**

The MDT is a reliable, valid, and easy-to-use instrument to rapidly measure the real-time moral distress of registered nurses in China.

## Introduction

Moral distress frequently challenges professional nurses when they provide care for their patients daily [1]. A series of instruments are currently available for measuring the perceived level of moral distress among healthcare professionals [2]. The moral distress scale-revised (MDS-R), which was developed by Hamric et al. [3]. based on the adaption of the initial MDS [4], was the most recognized one [2]. However, the MDS-R evaluates the level of moral distress by considering both frequency and intensity, making the use of this scale complex and time-consuming [5]. The MDT is a single-item instrument developed by Wocial and Weaver [5], which has been widely used to quickly evaluate real-time moral distress and accurately track its changes over time [6]. However, it remains unclear whether the MDT was suitable for Chinese cultural context.

## Background

Healthcare professionals frequently encounter ethical conflicts in their daily work due to repeated exposure to a large number of patients and their deaths [7]; however, compared with other professionals, professional nurses face higher risk of ethical conflicts because they are often outposts that provide most medical care to patients [8]. Therefore, the nursing is actually regarded as an “ethical laden practice” [9]. Moral distress may occur when professional nurses realize that they need to take morally correct action but their ability to execute such action is limited [10]. Moral distress has become one the most common moral sufferings that professional nurses frequently encountered in their daily care of patients, especially when they need to make rapid decisions [11]. Meta-analyses [1, 7] showed that the frequency and severity of moral distress in professional nurses are high and are a serious problem in professional nurses.

The undiscovered and unresolved moral distress will inevitably bring a series of negative consequences to professional nurses [12]. Studies have showed that moral distress is associated with increased stress, workplace fatigue, impaired professional relationships, job burnout, and poor professional identity, which can ultimately lead to an increased resignation rate of professional nurse [11, 13, 14]. In addition, under the negative impact of moral distress, professional nurses may unintentionally reduce their support for patients due to insufficient attention to patient’s suffering and avoidance of certain patient demands or needs, thereby affecting health outcomes [15]. Therefore, early and accurate detection of moral distress is critically important for reducing moral distress, thereby helping professional nurses to improve their nursing care quality [16].

In China, the issue of moral distress among healthcare professionals, particularly nurses, has garnered increasing recognition [13, 17, 18]. Chinese nurses frequently encounter unique ethical challenges due to the high patient-to-nurse ratios, cultural expectations, and systemic healthcare limitations [13, 17, 19]. These factors contribute significantly to a high level of moral distress, impacting their well-being, job satisfaction, career development, and the quality of care provided they provided [20–22]. For example, a study among Chinese nurses revealed that the pressure to conform to hierarchical decisions and the lack of autonomy significantly contribute to moral distress [23]. Additionally, the traditional cultural emphasis on collectivism and obedience can exacerbate feelings of helplessness and ethical conflict among Chinese nurses [17]. Addressing moral distress in this context is essential for improving both nurse retention and patient care outcomes.

Currently, several instruments have been developed to measure moral distress [2], such as the Ethical Stress Scale (ESS) [24], the Moral Distress Questionnaire (MDQ) [25] and the MDS [4]. Among these available instruments, MDS which was developed by Corley et al. [4]. has been the first one specific to intensive care nurses’ moral distress. Furthermore, Hamric et al. [3]. adapted the MDS to develop the MDS-R for use in healthcare professionals, reporting satisfactory internal reliability and construct validity. To date, the MDS-R has been adapted for use in different healthcare providers [26], such as adult-nurse, adult-physician, pediatric-nurse, and pediatric-physician, and it has also been cross-culturally adapted in different cultural contexts, such as Turkey [27], Italy [28], and Australia [29]. The MDS-R has also been culturally adapted for use in the Chinese cultural context in 2012 [30], and studies have used the Chinese version of the MDS-R to quantify moral distress and explore its influencing factors [23, 31]. Nevertheless, these available instruments are not without their limitations [5]. The first limitation revolves around the inherent cultural nuances in expressing distress or discomfort, which can introduce variability in the interpretation of scale items, therefore potentially undermining the reliability and validity of responses. The second limitation is that the practicality poses a significant challenge in Chinese healthcare settings. These comprehensive scales often require substantial time for completion, which is not conducive to the fast-paced and demanding nature of nursing work in China. With high patient volumes and intense workloads, nurse may find it burdensome to allocate the necessary time for through assessment. The most significant limitation is that the absence of specific time-point reference to differentiate cumulative moral distress from current moral distress. This deficiency impedes the accurate assessment of temporal trends and hampers the identification of time-sensitive interventions to alleviate moral distress among Chinese nurses.

The MDT was a single-item instrument, which was developed by Wocial and Weaver for rapidly measuring real-time moral distress of nurses [5]. By analyzing the data collected from 529 nurses practicing in hospital setting, these authors reported that the MDT achieved acceptable convergent validity and concurrent validity, therefore recommending it as a potential tool for screening the risk of moral distress and for evaluating the impact of interventions on moral distress. To date, many practitioners and researchers around the world have used MDT to measure the real-time moral distress in different healthcare providers and clinical settings, such as school nurses [32], ICU nurses [33], neonatal nurse [34] and oncology nurse [35], as well as community and hospital care [36, 37].

Nevertheless, to our knowledge, the MDT has not yet been introduced to China [2], thereby resulting in an open question about the applicability of the MDT in Chinese cultural context. Therefore, we performed this study aiming to translate the MDT to Chinese and adapt and validate the Chinese version of the MDT among the Chinese registered nurses. By introducing the original MDT to Chinese cultural context, our study not only addresses the limitations of existing tools in the Chinese cultural context but also offers tailored solutions to enhance the assessment of moral distress among Chinese registered nurses, ultimately contributing to the advancement of nursing practice, research, and education in China.

## Methods

### Aim

The aim of this study was to translate and culturally adapt the original MDT to Chinese, and evaluate the psychometrics of the Chinese version of the MDT in Chinese registered nurses.

### Study design

An online, cross-sectional, survey study of translating, culturally adapting, and validating MDT in the Chinese cultural context.

### Participants

Professional nurses legally registered in public hospitals in Chongqing municipal of China were eligible for inclusion. After the link of covering all questionnaires for survey was created, the principal investigator of this study shared the electronic link to the directors of nursing departments of targeted hospitals. Then, these directors forwarded this link to their working groups for the recruitment of participants. All working groups were created on WeChat application which is a popular social media platform in mainland of China. According to the Kendall sample estimation criteria [38], to assess the psychometric properties of instruments, the sample size should be at least five to seven times the number of items, with a minimum of 100 participants. Since MDT is a single-item instrument for measuring moral distress, we included 182 valid respondents in this validation study to ensure a reliable assessment of the measurement properties of the MDT.

### Instruments

#### Moral distress thermometer

The MDT is a tool designed by Wocial and Weaver in 2012 to rapidly and accurately measure real-time moral distress and track the trajectory of the moral distress over time [5]. The MDT is a thermometer-like rating tool, with 0 to 2, 2 to 4, 4 to 6, 6 to 8, and 8 to 10 indicating different levels of moral distress, corresponding to none, mild, uncomfortable, distressing, intense and worst possible, respectively. These authors of the original MDT designed an introduction to interpret the concept of moral distress and how to use this tool to mark their perceived level of moral distress: ‘Moral distress occurs when you believe you know the ethically correct thing to do, but something or someone restricts your ability to pursue the right course of action. Please circle the number (0–10) on the thermometer that best describes how much moral distress you have been experiencing related to work in the past 2 weeks, including today’ [5]. These authors used the MDS 2009 as the criterion tool to evaluate the convergent validity of the MDT, reporting that MDT had a low correlation with pediatric MDS 2009 and had a moderate correlation with adult MDS 2009. In addition, these authors also calculated the mean MDT scores among different turnover groups and compared the results with those results measured by the MDS 2009, indicating that MDT had satisfactory concurrent validity.

### Development of the Chinese version of the MDT

#### Translation

After obtaining the permission of the authors, two translators (X.T. and L.J.Y) translated the original MDT into Chinese following the key steps of the modified Brislin’s translation process, including a translation with back-translation [39]. The translation was done by two translators on the basis of fully considering both literal significance and context. Both translators, proficient in both Chinese and English and holding doctorates from the English doctoral program at the University of Rovira i Virgili, translated the text into their respective native languages, each translating in one direction. The individual performing the back translation is an experienced nursing teacher focusing on nursing ethics and has no idea about the original MDT. Next, the team of this study compared and discussed the translations until a consensus was reached, with the purpose of reaching operational similarity to the Chinese context.

#### Content validation

We built an expert panel to evaluate the content validity of the Chinese version of the MDT by assessing the relevance and comprehensiveness of the thermometer-like rating and the descriptive wordings [40]. Specifically, six experts with rich experience in clinical nursing, nursing ethics, and nursing management, including 2 registered nurses, 2 nursing educators, and 2 nursing administrators, reviewed and discussed the relevance and comprehensiveness of the Chinese version of the MDT in relation to content, semantics, criteria and conceptual nature of the thermometer-like rating and descriptive wordings. All experts invited to participant in the expert panel consistently gave feedback that “the descriptive wordings were accurate, and the rating on the thermometer is related to the experience of capturing moral resilience”, and “the introduction was clear and easy to understand, and descriptive words accurately clarified the MDT rating”.

#### Moral distress scale-revised

The Chinese version of the MDS-R which was validated by Sun et al. [30] based on the MDS-R developed by Hamric et al. [3], was used to as the criterion tool. The Chinese version of the MDS-R is a 22-item scale comprising the situations that cause moral distress in four subscales for “individual responsibility (8 items),” “not in the patient’s best interest (5 items),” “conflict of value (6 items),” and “damage patient’s interest (3 items).” Response to items measuring the frequency and intensity were rated on the 5-point Likert scale, with the rating scale ranging from 0 (never) to 4 (great extent). The score of moral distress was calculated by multiplying the scores of the frequency and intensity. The number of points of moral distress was calculated as the total score by adding all the scores for each question. A higher score indicated a stronger sense of moral distress. The Cronbach’s alpha coefficient in the initial version was 0.89, and the Chinese version of the MDS-R reported a Cronbach’s alpha coefficient of 0.88. In this study, a Cronbach’s alpha coefficient of 0.90 was obtained for the overall scale, with 0.85, 0.68, 0.69, and 0.72 for 4 subscales, respectively.

### Data collection

We created the link for questionnaire based on the *Sojump* (available at: https://www.wjx.cn/) which is the most popular online survey platform in mainland of China. The principal investigator of this study firstly built the collaboration relationship with the nursing directors of some hospitals in Chongqing municipal and then shared the link to these nursing directors for the purpose of collecting data using demographics collection form, MDT, and MDS-R. After these nursing directors informed the purpose and procedure of this study, they transferred the link to their working group for asking all professional nurse to finish the data collection.

### Statistical analysis

Descriptive statistics were used to describe demographic characteristics of participants. We used mean ± standard deviation (SD) to describe continuous outcomes because the violation of the normality assumption should not cause major problems when a large enough sample size was cumulated (> 30 or 40) [41]. Content validity was evaluated by an expert panel involving 6 experts through reviewing and discussing the relevance and comprehensibility of the Chinese version of the MDT. Convergent validity was evaluated by calculating the correlations between the MDT score and the scores of the MDS-R, which were indicated by Pearson’s correlation coefficients. Concurrent validity was evaluated by comparing: (1) the MDT scores of registered nurses working in different departments and (2) the MDT scores of registered nurses who represented different responses to the final questions in the MDS-R, ‘Have you ever left or considered leaving your working position due to moral distress’? - ‘No: I have never considered leaving or left’, ‘Yes: I have considered leaving my working position, but did not’ and ‘Yes: I have ever left a position’. A one-way ANOVA was used for these comparisons, and a Welch’s ANOVA was used if a homogeneity violation was found; however, a Games-Howell post-hoc test was used if a homogeneity was not violated. A *p* < 0.05 indicated statistical significance. IBM SPSS 26.0 software (SPSS Inc., Chicago, IL, USA) was used to perform data analysis.

### Ethical considerations

We performed this study in strict accordance with the Helsinki Declaration (2013), and the protocol of this study was approved by the Ethics Committee of Chongqing Traditional Chinese Medicine Hospital. All participants firstly signed the electronic informed consent before entering into the survey page. In addition, participants were informed that their participation in this study was entirely voluntary and would not have any consequences during the survey period due to withdrawal.

## Result

### Demographic characteristics of participants

A total of 182 respondents returned valid questionnaires for data analysis eventually, with an average age of 32.24 (± 5.98) years. Most of the respondents were males (79.1%), had married (67.6%) and bachelor’s degree (83.5%), experienced night shift (61.0%), and signed as contract nurses (65.4%). The detailed demographics of all participants are documented in Table 1.

**Table 1:** Demographics of 182 respondents returned valid questionnaires

Variables	Frequency	Percentage (%)
Sex		
Male	38	20.9
Female	144	79.1
Birth place		
Rural	128	70.3
Urban	54	29.7
Marital status		
Married	123	67.6
Unmarried	59	32.4
Educational level		
Associate bachelor degree or below	20	11.0
Bachelor’s degree	152	83.5
Master’s degree or above	10	5.5
Employment type		
Contract nurse	119	65.4
Permanent nurse	63	34.6
Job title		
Junior	89	48.9
Moderate	78	42.9
Senior	15	8.2
Night shift		
Yes	111	61.0
No	71	39.0
Monthly income		
2000–5000	23	12.6
5001–8000	89	48.9
> 8000	70	38.5
Working department		
Emergency	12	6.6
Internal medicine	19	10.4
Surgical medicine	7	3.8
Pediatric	11	6.0
Gynecological	1	0.5
ICU	7	3.8
Oncological	6	3.3
Anesthesiologic	5	2.7
Others	114	62.6

ICU, intensive care unit

### Score of moral distress

**Fig. 1:**
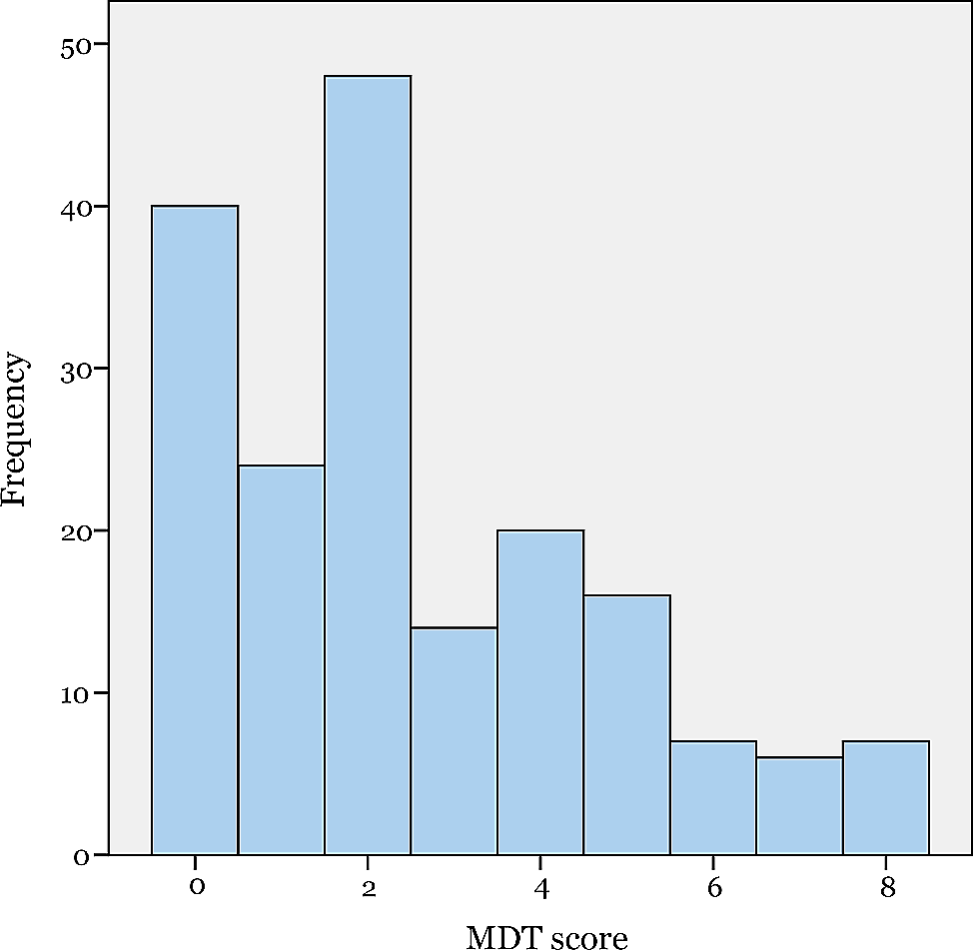
Distribution of the MDT scores of 182 participants

The result showed 3 outliers assessing a score of 10, and after omitting these outliers, a mean MDT score of 2.54 (± 2.21, 95% CI: 2.22–2.86) was calculated. We graphically the distribution of MDT scores among all participants, which was depicted in Fig. 1. The overall mean moral distress score measured by using the MDS-R was 38.66 (± 36.08, 95% CI 33.39–43.94), with an average frequency and intensity of 0.92 and 0.88, respectively.

### Content validity

All experts returned consistent feedbacks that the descriptive words in translation are relevant to rate moral resilience, and the introduction in translation clearly informs participants on how to use the tool. By referring the method to calculate the content validity index (CVI), the item-level CVI (I-CVI) and scale-level CVI (S-CVI) of descriptive words were both 1.000, indicating that the Chinese version of MDT has achieved satisfactory content validity.

### Convergent validity

We selected the MDS-R as the criterion tool to evaluate the convergent validity of the MDT. As presented in Table 2, the correlation coefficients of the MDT score with the total score of the MDS-R and the scores of its 4 subscales were 0.41, 0.46, 0.36, 0.31, and 0.42, respectively.

**Table 2:** Correlation between the MDT and the MDS-R

Variables	1	2	3	4	5	6
1. Mean MDT score	1.00					
2. Total score of MDS-R	0.41^**^	1.00				
3. Score in ‘individual responsibility’	0.46^**^	0.77^**^	1.00			
4. Score in ‘not in patient’s best interest’	0.36^**^	0.90^**^	0.61^**^	1.00		
5. Score in ‘conflict of value’	0.31^**^	0.93^**^	0.58^**^	0.79^**^	1.00	
6. Score in ‘damage patient’s interest’	0.42^**^	0.77^**^	0.71^**^	0.65^**^	0.63^**^	1.00

^**^indicates *p* < 0.01. *r* of ranging from 0.0 to 0.2, from 0.2 to 0.4, from 0.4 to 0.6, from 0.6 to 0.8, and from 0.8 to 1.0 indicates very weaken, weaken, moderate, high, and very high correlation, respectively. MDT, moral distress thermometer; MDS-R, moral distress scale-revised

### Concurrent validity

The result of the Welch’s ANOVA showed that registered nurses working different departments reported different levels of moral distress (F = 4.22; df = 7, 22.19; *p* = 0.004), and the Games-Howell post-hoc analysis revealed that registered nurses working in ICU reported the highest level of moral distress than those working in other departments (*p* = 0.04). In addition, result showed difference in the mean MDT scores of registered nurses who presented different responses to the final question of the MDS-R (F = 13.94; df = 2, 13.63; *p* = 0.001), and results of the Games-Howell post-hoc analysis further showed that registered nurses who had left (mean score = 5.17) and those who had considered leaving but had not left (mean score = 3.34) reported significantly higher MDT scores than those who had never consider leaving (mean score = 1.94), but there was no significant difference between those who had left and those who had considered leaving but had not left (*p* = 0.153).

## Discussion

The MDT has been wildly used for measuring real-time moral distress and tracking the changes of moral distress over time [6]; however, this tool is still not evaluated in Chinese cultural context. In this study, we translated the original version into Chinese, and evaluated its psychometric properties based on a sample of 466 Chinese registered nurses. The results show that the Chinese version of the MDT has good content and construct validities, therefore is suitable to rapidly and reliably measure moral distress of Chinese registered nurses and easily track the trajectory of moral distress over time.

To establish content validity of the Translated MDT, we employed a meticulous translation process. Specifically, two independent translators who are proficient in both Chinese and English rendered the original MDT into their representative languages by meticulously considering both literal meaning and cultural context. The research team then convened to achieve consensus on a final Chinese version. Subsequently, a 6-member expert panel reviewed and discussed the relevance and comprehensibility of the Chinese version of the MDT. The consistent feedback throughout this process indicated that the translated MDT utilizes clear, relevant descriptive words to rate moral distress. Additionally, the introduction provides participants with readily understandable instructions for complete the tool. These results collectively demonstrate that the Chinese version of MDT possesses strong content validity for application among Chinese registered nurses.

This study also evaluated the criterion validity of the Chinese version of MDT by referring the MDS-R [30]. The Chinese version of MDS-R has satisfactory reliability, with a Cronbach’s alpha coefficient of 0.88. So, it is reasonable and reliable to evaluate the Chinese version of the MDT by using the Chinese version of the MDS-R as the criterion validity index. The result of Pearson correlation analysis showed that the Chinese version of the MDT had low to moderate correlation to the MDS-R and its 4 subscales (*p* < 0.01), implying that the Chinese version of the MDT did measure moral resilience of Chinese registered nurse. Considering that MDS-R consists of abundant items and complex response to items, the MDT may be more suitable for evaluating the real-time moral distress and tracking the changes of moral distress in intervention study [42–44].

Previous studies have shown that departments with more decisions regarding ethical issues (e.g., ICU and emergency) may cause more moral conflicts than those that had higher acuity and lower predictability [45, 46]. Jawed et al. [47]. reported that the perceived moral distress varied by specialty, and critical care had more moral distress than specialties labeled as “other”. Moreover, the study performed by Wolf et al. [48]. revealed that moral distress in ICU was higher than other departments because sources of moral distress are more frequent and intense with conflicting goals, more infliction of harm, pain and suffering. In our study, registered nurses’ perceived level of moral distress was compared by different departments, and the results showed that those working in ICU reported the highest degree of moral distress than those who worked in “other” departments, which is consistent with findings of previous studies, indicating a good discriminant validity of the MDT.

We also evaluated the concurrent validity of the Chinese version of the MDT by comparing the MDT scores between registered nurses who made different responses to the final question of the MDS-R. The results showed that registered nurses who had ever left and those who had considered leaving but had not left rated higher MDT scores than those who had never consider leaving. In their study, Wolf et al. [48]. reported that the MDT scores of participants who currently intended to leave their working positions are significantly higher than those of other participants [48]. In addition, when evaluated the psychometrics of the Swedish version of the MDT, Grönlund et al. [6]. also consistently found that healthcare professionals who had ever left and those who had considered leaving but had not left reported significantly higher moral distress than those who had never considered leaving their position. All these findings were consistent with our study, suggesting that the Chinese version of the MDT had acceptable concurrent validity and also implying that leaving the current working position seems to be one way for registered nurses to effectively address moral distress. According to the Rushton’s theoretical framework [49], unaddressed moral distress may impair an individual’s wellbeing and cause moral harm, further resulting in outrage and professional burnout; however, avoidance and abandonment can be ways to transparently respond to such suffering.

This study has three limitations. The first one is that we did not evaluate the reliability of the Chinese version of the MDT by designing the test-retest trial. However, as stated by Wocial and Weaver in their instrument development study [5], moral distress is a changeable variable over time, therefore it may not be suitable to evaluate the test-retest reliability of the MDT. Second, the sample size of 182 registered nurses met the minimum criteria but did not reach the maximum criteria, therefore we suggest future studies also evaluate the psychometrics of the Chinese version of the MDT when they use this tool to measure moral distress. Third, we recruited participants through online platform, which inevitably undermines the robustness of our results due to sample bias.

## Conclusion

The study successfully adapted and validated the MDT for use among Chinese nurses, demonstrating its relevance and effectiveness in measuring moral distress in this population. Overall, the Chinese version of the MDT offers a practical and valuable instrument for assessing moral distress, paving the way for better support and management strategies for nurses in China.

### Relevance to clinical practice

The Chinese version of the MDT provides an accurate and reliable tool for measuring the perceived level of moral distress among registered nurses. The acceptable criterion and concurrent validity of the translated MDT ensure that it reflects the nurses’ experiences accurately, making it an essential tool for healthcare administrators and practitioners. By utilizing the MDT, healthcare institutions can systematically assess the moral distress levels of their nursing staff, identify areas of concern, and implement targeted interventions to address these issues. The ability to reliably measure moral distress allows for timely and effective strategies to mitigate its negative impact, thereby enhancing the overall well-being of nurses and improving patient care outcomes. Additionally, the MDT’s ease of use facilitates regular monitoring and evaluation, ensuring that any changes in moral distress levels are promptly addressed, fostering a supportive and resilient clinical environment.

### Relevance to future research

The validated Chinese version of the MDT provides new avenues for future research in nursing and healthcare. Researchers now have a robust and validated instrument to measure moral distress among Chinese registered nurses, which can be employed in various studies to easily and simply explore the prevalence, influencing factors, and consequences of moral distress in this population. Future research can use the MDT to evaluate the effectiveness of different interventions designed to alleviate moral distress, providing empirical evidence on the most effective strategies for supporting nurses. Longitudinal studies can use the MDT to track changes in moral distress over time, offering insights into the long-term impact of institutional policies and practices on nurses’ moral well-being. Furthermore, comparative studies could employ the MDT to explore differences in moral distress across different cultural contexts or healthcare systems, contributing to a more comprehensive understanding of this phenomenon. The availability of the MDT thus lays the groundwork for a wide range of research endeavors aimed at improving the work environment and mental health of nurses, ultimately enhancing the quality of patient care.

## Data Availability

The data are available from the corresponding author on reasonable request.
